# Psychological risk factors for Long COVID and their modification: study protocol of a three-arm, randomised controlled trial (SOMA.COV)

**DOI:** 10.1192/bjo.2023.591

**Published:** 2023-11-03

**Authors:** Petra Engelmann, Christian Büchel, Jördis Frommhold, Hans F. E. Klose, Ansgar W. Lohse, Kerstin Maehder, Yvonne Nestoriuc, Martin Scherer, Anna Suling, Anne Toussaint, Angelika Weigel, Antonia Zapf, Bernd Löwe

**Affiliations:** Department of Psychosomatic Medicine and Psychotherapy, University Medical Center Hamburg-Eppendorf, Germany; Institute of Systems Neuroscience, University Medical Center Hamburg-Eppendorf, Germany; Institute Long Covid, Rostock, Germany; II. Medical Clinic and Polyclinic, University Medical Center Hamburg-Eppendorf, Germany; I. Department of Internal Medicine, University Medical Center Hamburg-Eppendorf, Germany; Institute of Systems Neuroscience, University Medical Center Hamburg-Eppendorf, Germany; and Department of Psychology, Helmut Schmidt University, Germany; Department of General Practice and Primary Care, University Medical Center Hamburg-Eppendorf, Germany; Institute of Medical Biometry and Epidemiology, University Medical Center Hamburg-Eppendorf, Germany

**Keywords:** Long COVID, post-COVID-19 condition, persistent somatic symptoms, biopsychosocial model, risk factors

## Abstract

**Background:**

Growing evidence suggests that in addition to pathophysiological, there are psychological risk factors involved in the development of Long COVID. Illness-related anxiety and dysfunctional symptom expectations seem to contribute to symptom persistence.

**Aims:**

With regard to the development of effective therapies, our primary aim is to investigate whether symptoms of Long COVID can be improved by a targeted modification of illness-related anxiety and dysfunctional symptom expectations. Second, we aim to identify additional psychosocial risk factors that contribute to the persistence of Long COVID, and compare them with risk factors for symptom persistence in other clinical conditions.

**Method:**

We will conduct an observer-blinded, three-arm, randomised controlled trial. A total of 258 patients with Long COVID will be randomised into three groups of equal size: targeted expectation management in addition to treatment as usual (TAU), non-specific supportive treatment plus TAU, or TAU only. Both active intervention groups will comprise three individual online video consultation sessions and a booster session after 3 months. The primary outcome is baseline to post-interventional change in overall somatic symptom severity.

**Conclusions:**

The study will shed light onto the action mechanisms of a targeted expectation management intervention for Long COVID, which, if proven effective, can be used stand-alone or in the context of broader therapeutic approaches. Further, the study will enable a better understanding of symptom persistence in Long COVID by identifying additional psychological risk factors.

Studies suggest that after an infection with SARS-CoV-2 has abated, a substantial portion of affected patients do not fully recover and may be at risk of persistent somatic symptoms – a phenomenon often described as ‘Long COVID’. There is growing evidence that the development of Long COVID is multifactorial and involves pathophysiological, psychological and social mechanisms.^1^ Among psychological risk factors, increased levels of illness-related anxiety^2–4^ and dysfunctional symptom expectations^5,6^ appear to contribute to processes of symptom persistence after SARS-COV-2 infection. Since both factors can potentially be modified by targeted interventions, this study will investigate a defined mechanism of action: whether Long COVID can be influenced by modifying illness-related anxiety and dysfunctional symptom expectations.

The term Long COVID has become widely used to describe persistent somatic symptoms after a SARS-CoV-2 infection has resolved. Although Long COVID syndrome still lacks specific definition, classification and diagnostic guidelines,^2^ it is thought to comprise symptoms that last >4 weeks after the onset of a SARS-CoV-2 infection, can affect almost every organ system in the body, may change over time and are not better explained by an alternative diagnosis.^7^ According to the UK National Institute for Health and Care Excellence (NICE) COVID-19 guideline and the German Association of the Scientific Medical Societies (AWMF) S1-guideline ‘Post-COVID/Long-COVID’, Long COVID subsumes both ‘ongoing symptomatic COVID-19’^8^ (4–12 weeks after infection) and ‘post-COVID-19 syndrome’ (>12 weeks after infection).^7^ Thus, Long COVID falls under the umbrella term persistent somatic symptoms (PSS), which is used to describe subjectively distressing somatic complaints, irrespective of their aetiology, that are present on most days for at least several weeks.^9^

## Clinical presentation of Long COVID

Long COVID is considered an unusual course of disease, as COVID-19 is usually expected to subside after 2 weeks for patients with mild manifestation, and up to a maximum of 6 weeks for those with severe disease.^10^ It is associated with reduced quality of life and poses challenges to the healthcare system, e.g. through work absenteeism and increased healthcare use. According to a meta-analysis of 15 studies encompassing 47 910 patients, 80% stated at least one symptom during follow-up periods ranging from 2 weeks to 4 months post-infection.^11^ In a German prospective cohort study, only 22.9% of patients indicated to be free of symptoms at 12 months after symptom onset.^12^ The most frequently reported symptoms at 12 weeks in a prospective study of 4182 COVID-19 cases were fatigue, shortness of breath, loss of smell and headache.^1^ A NICE rapid evidence review of three high-quality systematic reviews also found, among others, high mean percentages for fatigue (31–51%), dyspnoea (22–38%), impairment of smell (15–24%) and taste (7–16%), and muscle pain (5–22%) after at least 4 weeks.^7^

## General aetiological model for PSS

Since there is no generally accepted disease-specific model for the development of Long COVID, we draw on disease-overarching models for the development of PSS by analogy. Regarding the transition from short-term to persistent disabling symptoms in general, the comprehensive vulnerability-stress model by Henningsen et al^13^ defines predisposing, triggering and maintaining/aggravating factors that determine the transition from short-term symptoms to PSS. Based on this model and current research findings, the German Research Foundation (DFG)-funded interdisciplinary SOMACROSS Research Unit (Persistent Somatic Symptoms Across Diseases: From Risk Factors to Modification; unit identifier RU 5211), which investigates generic and disease-specific risk factors and mechanisms for the development and maintenance of PSS across ten medical conditions, developed a cross-disease working model for the aetiology of PSS. The model is described in detail in the published framework and overarching protocol of the research unit.^9^ In brief, according to this model, predisposing factors for PSS comprise sociodemographic (e.g. female gender, poor education), psychological (e.g. precedent life stressors, negative affectivity) and biomedical risk factors (e.g. prior medical conditions, immunological predispositions). Triggering factors for short-term somatic symptoms include acute infections, injuries or current life stressors. Among the best-established maintaining/aggravating factors for PSS is illness-related anxiety,^14^ which may be explained by its association with other cognitive–perceptual and emotional mechanisms (e.g. catastrophising, somatosensory amplification) and with behavioural processes (e.g. avoidance behaviour). Besides disease-specific (e.g. inflammation, organ damage) and overarching (e.g. treatment effects) biomedical factors, dysfunctional symptom expectations are considered highly relevant for symptom persistence.^9^ Symptom expectations are defined as conscious, future-directed cognitions regarding the anticipated course of symptoms.^15^ As they constitute a common denominator of many psychological risk factors for PSS, expectations are regarded as a core feature of current aetiological models for PSS.^16^ They are also prominently conceptualised in new predictive processing models suggesting that symptom perception emerges through an unconscious, integrative process of sensory input, contextual cues and prior information. In these models, next to previous experiences, learning processes, context and prior knowledge, expectations are considered one form of prior information that contribute to the development of implicit predictions about the presence of symptoms.^17^

## General risk factors for Long COVID

In terms of general risk factors for developing Long COVID, older age, female gender, obesity, viremia, specific autoantibodies and pre-existing medical conditions have been found.^1,11,18^ However, according to current evidence, a substantial number of low-risk patients with COVID-19 report ongoing somatic symptoms months after the infection.^19^ Therefore, it seems reasonable to assume that further clinical features are associated with the subsequent development of Long COVID. For example, in a recent case–control study, 443 individuals showed signs of modest subclinical multi-organ dysfunction after mild-to-moderate SARS-CoV-2 infection.^20^ Although there is no doubt about the importance of disease-specific pathophysiological processes, no biomarker has yet been identified to comprehensively measure the severity of Long COVID. A reason to believe that pathophysiological damage is not the only contributor to Long COVID symptoms is that several studies indicated a small or no association between Llong COVID and initial disease severity.^1^ Two particularly relevant and potentially modifiable risk factors for Long COVID are discussed below.

## Anxiety as a risk factor for Long COVID

In a retrospective electronic health record study including 236 379 individuals, Taquet et al^2^ examined neurologic and psychiatric symptoms within the first 6 months after COVID-19 diagnosis. The highest incidence was found for anxiety disorders at 17.4%, of whom 10% had already been diagnosed before their SARS-CoV-2 infection. A recently published cohort study of 3193 individuals found anxiety and worry about COVID-19, as well as depression, perceived stress and loneliness, to be prospectively associated with an increased risk of self-reported Long COVID.^3^ Further supporting the assumption that illness-related anxiety is involved in the development and maintenance of Long COVID, we found anxiety to be the strongest modifiable predictor of somatic symptom burden after 8 weeks in a prospective cohort study of 1185 healthcare professionals that did not contract COVID-19.^4^ In the UK general population, COVID-19-related anxiety has also been found to predict somatic symptoms, particularly fatigue.^21^ Consistent with these findings, illness-related anxiety has been found to be a risk factor for PSS before the COVID-19 pandemic in a range of medical conditions, e.g. chronic fatigue, fibromyalgia and irritable bowel syndrome.^14^

## Expectations as a risk factor for Long COVID

In a recently concluded prospective cohort study of German adults, we assessed risk factors for somatic symptom deterioration after 21 months (*N* = 751). Symptom expectations associated with COVID-19 and a self-reported history of COVID-19, but not serologically confirmed SARS-CoV-2 infection, significantly predicted somatic symptom deterioration 21 months later.^5^ Our findings corroborate the results of a cross-sectional population-based French cohort study including 26 823 participants, which also indicated that PSS after COVID-19 may be more strongly associated with belief in having been infected with SARS-CoV-2 than with a laboratory-confirmed infection.^6^ Consequently, the expectation of symptoms could be a determining factor for symptom persistence in Long COVID. Similarly, patients’ expectations of symptom severity have been found to be a crucial factor in other conditions, e.g. chronic fatigue.^22^ The power of expectations to predict symptom course, treatment benefit and negative treatment side-effects has been demonstrated for a wide range of medical illnesses and somatic symptoms, such as cancer, pain, gastrointestinal and ‘medically unexplained’ symptoms.^23,24^

## Specific aetiological model for persistent Long COVID symptoms

**Fig. 1:**
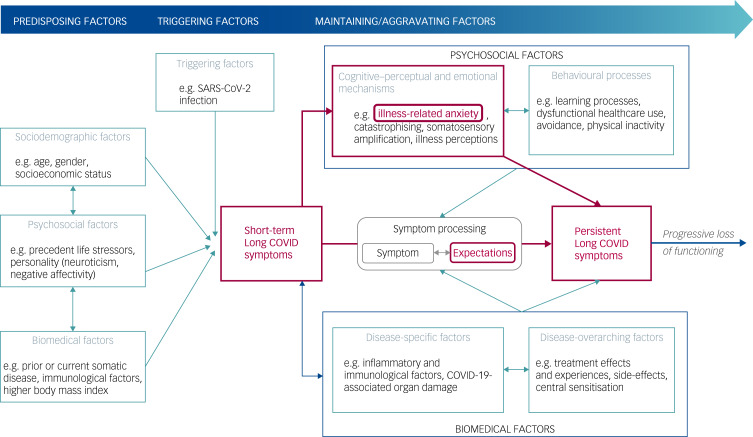
General model for the aetiology of persistent somatic symptoms, developed by the SOMACROSS Research Unit^9^ and adapted for Long COVID. The factors specifically investigated in this study are marked in red.

The above described risk factors for Long COVID are congruent with the risk factors described in the cross-disease SOMACROSS model for the aetiology of PSS.^9^ Thus, we assume that this cross-disease biopsychosocial model for symptom persistence might also be applicable to Long COVID. Therefore, we adapted the working model of the SOMACROSS Research Unit by entering predisposing, triggering and maintaining/aggravating factors assumed for Long COVID (see Fig. 1).

## Research needs

Empirical evidence points to a multifactorial biopsychosocial model regarding the aetiology of Long COVID. Illness-related anxiety and dysfunctional symptom expectations appear to be relevant modifiable factors contributing to Long COVID independent of initial disease severity. Thus, modifying illness-related anxiety and expectations in patients with Long COVID may be a promising approach in improving symptoms. A recent search in PubMed and the World Health Organization (WHO) International Clinical Trials Registry Platform (ICTRP) indicated that so far, no study has investigated alleviating Long COVID symptoms by targeting these factors. Should a targeted modification of illness-related anxiety and dysfunctional expectations of symptom severity lead to a change in Long COVID symptoms, this would provide sound evidence for a specific mechanism of action for the development of Long COVID and the effectiveness of a mechanism-based treatment approach.

## Objectives and hypotheses

### Objectives

Objective 1 is to investigate whether overall somatic symptom severity in patients with Long COVID can be improved via the modification of illness-related anxiety and dysfunctional symptom expectations. Objective 2 is to prospectively identify further risk factors involved in the persistence of Long COVID, and to deduct conceptual models of symptom persistence, deterioration and improvement. Objective 3 is to compare risk factors and mechanisms of somatic symptom persistence in Long COVID exploratively with those identified in the medical conditions investigated in the SOMACROSS Research Unit.

For objective 1, a testable hypothesis can be formulated. Objective 2 will be investigated with an exploratory approach. To address objective 3, we will use data from the SOMACROSS Research Unit and compare them with our project data in an exploratory manner.

### Hypotheses

Hypothesis 1 is that the therapeutic modification of illness-related anxiety and dysfunctional symptom expectations improves Long COVID symptom severity. Hypothesis 2 (exploratory) is that, in addition to illness-related anxiety and dysfunctional symptom expectations, further risk factors contributing to the persistence of Long COVID symptoms can be identified. Hypothesis 2 (exploratory, using results from SOMACROSS) is that Long COVID and other medical conditions share common risk factors for somatic symptom persistence.

## Method

### Study design

#### Study design and rationale

To identify the effect of a targeted modification of illness-related anxiety and dysfunctional symptom expectations on Long COVID symptoms, and to differentiate this effect from general modes of action, a randomised comparison between a specifically treated group, a group treated non-specifically in the same dose and a control group without additional treatment is required. A control group is necessary to test whether the experimental interventions have a positive effect compared with no intervention at all, and to investigate objectives 2 and 3. Thus, we will use the design of a three-arm randomised controlled trial, in which one third of participants will undergo targeted expectation management in addition to treatment as usual (TAU), one third will undergo unspecific supportive treatment in addition to TAU, and one third will receive TAU only. In the control group, we will additionally investigate the contribution of predefined risk factors to symptom persistence in Long COVID. The study will be fully observer-blinded with respect to all treatment conditions. It will be conducted monocentric with nationwide recruitment.

#### Setting

For recruitment, we will use our pulmonary out-patient clinic as well as our psychosomatic out-patient clinic and our established network of general practitioners and pulmonologists. We will also recruit via social media campaigns with support of cooperating self-help groups and patient organisations (e.g. ‘Landesverband Sachsen für Prävention und Rehabilitation von Herz-Kreislauf-Erkrankungen e.V. ‘, ‘Long COVID Vernetzungsstelle’, ‘Nationale Kontakt- und Informationsstelle zur Anregung und Unterstützung von Selbsthilfegruppen’). The experimental interventions will be carried out as personal online video consultations, which allows for a nationwide outreach of our study.

#### Inclusion criteria

Inclusion criteria are as follows: self-reported resolved SARS-CoV-2 infection confirmed by a positive polymerase chain reaction, serology or rapid antigen test; presence of Long COVID according to the NICE/AWMF S1-guidelines,^7^ at least moderately severe ongoing symptoms (Patient Health Questionnaire 15 (PHQ-15) score ≥10),^25^ aged ≥18 years and provision of informed consent.

#### Exclusion criteria

Exclusion criteria are as follows: acute SARS-CoV-2 infection, intensive care unit treatment for COVID-19, psychotherapeutic treatment in the past 3 months, necessity of acute emergency treatment, acute suicidality, a substance use disorder, acute psychosis, cognitive incapacity to comprehend the study materials, inability to complete outcome measures online or insufficient German language skills.

### Experimental interventions and control intervention

#### Experimental intervention 1 (COV.EXPECT ± TAU)

The experimental intervention consists of an expectation management intervention (COV.EXPECT) in addition to TAU. The manualised intervention primarily aims to reduce illness-related anxiety and optimise expectations about symptoms, treatment outcome and coping strategies.^26^ The design and dose of the intervention are based on the demonstrated effectiveness of the expectation management intervention from the PSY-HEART trial (Psychological Preoperative Interventions to Improve Outcome in Heart Surgery Patients),^27^ on the SOMA. GUT study (Persistence of Gastrointestinal Symptoms in Irritable Bowel Syndrome and Ulcerative Colitis: From Risk Factors to Modification) within the SOMACROSS Research Unit^28^ and on other previous studies.^26^ The intervention consists of three individual online video consultation sessions (conducted fortnightly) and a booster session after 3 months, with each session lasting 45 min. In the first session, the patient's expectations regarding symptoms and treatment will be assessed through a semi-structured interview so that the intervention can be adapted to the individual patient expectations within the framework of the treatment manual. The intervention components include psychoeducation aimed at developing a biopsychosocial model of Long COVID and realistic, yet functional expectations regarding symptoms and treatment outcome, visualisation techniques to foster expectations of personal control and developing personal goals in managing symptoms to improve coping expectations. In a ‘toolbox’, illness-specific dysfunctional expectations (e.g. ‘I can't take the stairs because I'm too short of breath’) are assigned to specific therapeutic interventions (e.g. cognitive restructuring, fostering positive expectations via visualisation and addressing confidence in expectations, counteracting cognitive immunisation). To deepen the acquired skills, homework will be given after each session and discussed at the beginning of the following one. The intervention thus addresses the topics ‘dealing with anxiety’, ‘improving expectations’ and the need for information about their disease. By conducting focus groups, the intervention protocol and manual will be finalised in collaboration with patients from a Long COVID group currently offered in our psychosomatic out-patient clinic.

#### Experimental intervention 2 (COV.SUPPORT ± TAU)

The experimental intervention consists of a non-specific supportive intervention (COV.SUPPORT) in addition to TAU. COV.SUPPORT is identical to COV.EXPECT in terms of common and non-specific treatment elements (i.e. time, personal attention and emotional support), but does not use specific interventions to modify illness-related anxiety and expectations. In contrast to COV.EXPECT, which focuses primarily on changing dysfunctional symptom expectations for the future, COV.SUPPORT focuses exclusively on coping with stressful situations in the present. COV.SUPPORT is manualised and adapted from the supportive therapy we use in the PSY-HEART-II trial.^29^ This unspecific intervention is needed to differentiate our proposed mechanism of action from general modes of action.

#### Control intervention (TAU)

The control intervention consists of TAU only. TAU in all study groups implies that the patient receives their usual treatment without any interference by the study. This group is also needed to identify risk factors for the persistence of Long COVID symptoms (objective 2) and compare risk factors for symptom persistence across conditions with results from the SOMACROSS Research Unit (objective 3).

### Assessment and study outcomes

#### Measurement points

Assessments are carried out at baseline and after 6 weeks (intermediate), 3 months (post-interventional) and 6 months (follow-up). The intermediate assessment after 6 weeks is necessary for mediator analyses that investigate whether a change in symptom severity is mediated via changes in illness-related anxiety and dysfunctional symptom expectations. Except for an additional 12-month follow-up in the SOMACROSS Research Unit, assessment times are identical, allowing for comparison of results. Primary and secondary outcomes and mediator variables will be collected through electronic data entry by patients at home; only the diagnostic interview will be collected via telephone interview. The primary and secondary outcome variables and mediator variables will be measured at each assessment point, and are displayed in Table 1.

**Table 1:** Outcome instruments of the SOMA.COV study

Outcome variables (assessed via self-report/diagnostic interview)						
Predisposing, triggering and maintaining/aggravating factors	Single constructs	Instrument	Assessment time			
			0 months	6 weeks	3 months	6 months
Primary outcome: somatic symptoms	Overall somatic symptom severity	Patient Health Questionnaire-15 (PHQ-15)	X	X	X	X
Secondary outcomes: symptom-related disability	SARS-CoV-2 infection and Long COVID	Single items	X	X	X	X
	Long COVID symptoms	Patient Health Questionnaire-15 Post-Acute Infection Syndromes (PHQ-15 PAIS)	X	X	X	X
	Fatigue	Fatigue Scale (FS)	X	X	X	X
	Post-exertional malaise	DePaul Symptom Questionnaire Post-Exertional Malaise (DSQ-PEM)	X	X	X	X
	Pain	Pain Disability Index – adapted (PDI)	X	X	X	X
	SOMACROSS core measures^9^	Self-report instruments	X	X	X	X
Diagnosis of somatic symptom disorder (DSM-5)	Diagnostic classification	Structured Clinical Interview for the DSM-5 (SCID-5)	X		X	

Prespecified mediator variables (assessed via self-report)						

Cognitive–perceptual and emotional mechanisms	Illness-related anxiety	Somatic Symptom Disorder – B Criteria Scale (SSD-12)	X	X	X	X
	Treatment expectations	Treatment Expectation Questionnaire (TEX-Q)	X	X	X	X
	Expectation of symptom severity	Numeric Rating Scale (NRS)	X	X	X	X
	Expectation of symptom burden	Numeric Rating Scale (NRS)	X	X	X	X
	Expectation of coping with symptoms	Numeric Rating Scale (NRS)	X	X	X	X

#### Primary outcome

To test the effect of the expectation management intervention on Long COVID symptoms, the primary outcome for this study is the baseline to post-interventional change in overall somatic symptom severity (3-month follow-up). As harmonised core outcome sets for Long COVID and post-COVID-19 conditions are currently still being developed, overall symptom severity will be assessed with the established PHQ-15,^25^ which is validated in English and German in various diseases and has proven its sensitivity to change as an outcome instrument in many studies.^30^ On a scale of 0 (‘not bothered at all’) to 2 (‘bothered a lot’), the PHQ-15 uses 15 items to measure somatic symptom severity during the past 4 weeks. It includes most of the common Long COVID symptoms and enables us to compare our results to the SOMACROSS Research Unit, which also uses the PHQ-15 as a primary outcome instrument.^9^

#### Secondary outcomes

Secondary outcomes include symptoms that have been found to be of special relevance in Long COVID, such as fatigue,^31^ post-exertional malaise^32^ and pain.^33^ We will also include a self-developed screening questionnaire on Long COVID as well as other post-infectious symptoms. This self-report instrument, called the PHQ-15 Post-Acute Infection Syndromes (PHQ-15 PAIS), is based on the PHQ-15^25^ and extended for the most common symptoms in post-acute infection syndromes.^34^ Illness-related anxiety (Somatic Symptom Disorder – B Criteria Scale (SSD-12)),^35^ expectations of symptom severity, burden, treatment outcome and coping with symptoms (Treatment Expectation Questionnaire (TEX-Q); European Research Network to Improve Diagnosis, Treatment and Health Care for Patients with Persistent Somatic Symptoms (EURONET-SOMA) numeric rating scales)^36,37^ will be investigated as pre-specified mediator variables. The treatments received will be documented. In addition to Long COVID-specific outcome variables, we will apply the joint core measures of the SOMACROSS Research Unit to identify further risk factors and mechanisms for symptom persistence in Long COVID, and to make the results of the studies comparable. The joint core set of instruments is described in detail in the overarching protocol of the SOMACROSS Research Unit.^12^ Supplements from the core set include, for example, adverse childhood experiences, negative affectivity, stigmatisation, health-related quality of life and healthcare utilisation. As in the research unit, we will also conduct the Structured Clinical Interview for the DSM-5 (SCID-5) for the diagnosis of somatic symptom disorder according to the DSM-5, to investigate the diagnosis as a potential predictor of treatment response.

#### Safety outcomes

To the best of our knowledge, there is no risk for serious adverse events caused by the application of expectation management interventions.^27^ However, patients may develop severe somatic complications of Long COVID or other medical conditions. In these cases, patients will be informed and advised to initiate appropriate treatment. In case of an emergency, medical treatment will be initiated at the University Medical Center Hamburg-Eppendorf. Any adverse events will be systematically recorded at each follow-up assessment.

#### Sample size

**Fig. 2:**
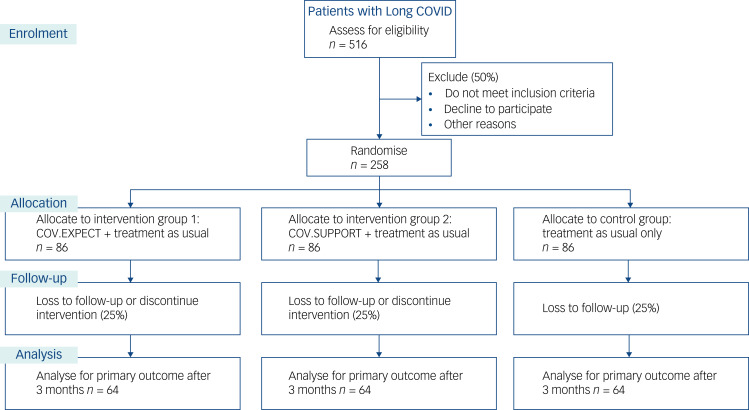
Anticipated flow of participants through the course of the study. Outcomes after 6 months are secondary and were not included in the sample size estimation. COV.EXPECT, expectation management intervention; COV.SUPPORT, supportive intervention.

This trial is powered with regard to the difference between intervention 1 (COV.EXPECT + TAU) and the control condition (TAU), as this is the main interest. Because of the closed testing principle, the type one error (alpha) does not need to be adjusted for multiplicity. As there are no comparable trials on Long COVID available, we based our estimation both on our DEPSCREEN-INFO trial (Increasing the Efficiency of Depression-Screening using Patient-Targeted Feedback),^38^ which yielded a between-group effect size of *d* = 0.33 in PHQ-15 scores with an intervention of much lower intensity (written feedback after depression screening), and on the PSY-HEART trial,^27^ which yielded a medium between-group effect size of *d* = 0.71 with an expectation management intervention, but using a different outcome instrument (modified version of the Pain Disability Index). Based on these two studies, we expect a medium effect size (*d* = 0.50) that can be detected with a power of 80%, using a two-sided alpha of 5%, by including 64 patients in each group, resulting in a total sample size of *N* = 192. Based on the results of our previous studies, we assume a loss to follow-up between baseline and the primary outcome measurement (i.e. 3-month follow-up) of 25%, resulting in a total of 258 randomised patients. Assuming that 50% of patients will meet the inclusion criteria, a total of 516 patients will be assessed for eligibility. Figure 2 shows the anticipated flow of participants throughout the trial.

#### Statistical methods

The primary analysis and all prespecified secondary analyses will be conducted in the intention-to-treat sample consisting of all randomised patients with the full analysis set, which is as close as possible to the intention-to-treat population. In secondary analyses, the data of the per-protocol population (subgroup of patients without major protocol violations) will be used and multiple imputation will be applied.

For objective 1, a linear mixed-effects model with the change from baseline for all measured time points will be used to investigate the group differences in the PHQ-15, with treatment group, time and gender as fixed effects, patient as the random effect and baseline PHQ-15 score as the covariate. The interaction between time and treatment group will be tested and excluded from the model if *P* ≥ 0.05. For primary hypothesis, the global treatment effect after 3 months will be considered first. If the two-sided *P*-value is <0.05, the contrasts in change after 3 months for COV.EXPECT versus TAU, COV.SUPPORT versus TAU and COV.EXPECT versus COV.SUPPORT at the 5% level (two-sided hypothesis) will be considered. Because of the closed testing principle, an adjustment of the contrast *P*-values for multiplicity is not necessary.

Secondary end points will be analysed analogously to the primary end point, with the regression model corresponding to the measurement scale (mixed linear model for metric outcomes, mixed logistic model for binary outcomes, cumulative logit model for ordinal outcomes).

In subsidiary analyses, a potential effect of recruitment settings will be investigated by including the setting as a further fixed effect in the analysis. To analyse whether treatment effects on Long COVID resulted through changes in illness-related anxiety or symptom expectations, we will conduct mediation analyses.

To identify further risk factors involved in the persistence of Long COVID (objective 2), we will use longitudinal data from the control group and conduct multivariate regression analyses with symptom persistence as outcome, taking into account the number of predictors and sample size. Patients from the intervention groups will not be included in these analyses because the interventions could bias the results.

To compare risk factors and mechanisms of somatic symptom persistence in Long COVID exploratively with those identified in the other conditions investigated in the SOMACROSS Research Unit (objective 3), we will compare the results of the disease-specific regression analyses from the SOMACROSS Research Unit with the derived model from objective 2, and conduct further exploratory analyses.

Subgroup analyses will be conducted on differences in the primary outcome with respect to the following variables of interest: age, gender, education level, social support, duration and severity of Long COVID, any chronic physical disease, any mental health comorbidity, depression and anxiety. For safety analyses, adverse events will be compared between groups and analysed descriptively. Further details of statistical analyses will be described in detail in a statistical analysis plan, which will be finalised before breaking the treatment blind.

#### Methods against bias

A fixed randomisation list with variable block size and length, stratified by gender, will be generated by an independent member of the biostatistics unit and uploaded into REDCap version 13.7.7 for Windows (REDCap Consortium; see www.projectredcap.org), a software for building and managing online surveys and databases. Assignment will be performed automatically after inclusion in the database. Patient drop-out will be minimised by contacting patients according to a schedule of repeated contact attempts, and by allowing solely written or telephone data collection if electronic collection is not feasible. Because of the electronic collection of outcome variables and having the telephone interviews conducted by trained interviewers who are not involved in the treatment, the study is fully observer-blinded with respect to all treatment conditions. Treating physicians will not be informed about the group allocation or the type of intervention. As in most psychological intervention studies, full patient and therapist blinding is not feasible as their active involvement in the intervention is necessary. Still, patients in the active intervention groups will be blinded. Both interventions will be manualised and therapists and interviewers will be trained and supervised. As a manipulation check regarding potentially overlapping content, contamination and carry-over effects between the two interventions, patients will complete a rating scale on treatment content and on subjective treatment mechanisms after the post-intervention outcome assessment. Potential sampling bias will be avoided by recruiting patients both within and outside of psychotherapeutic care, and by aiming to include both patients with shorter and longer symptom duration in the study. Any questions regarding patient exclusions, serious adverse events and potential study termination will be reviewed by the study's Data Safety and Monitoring Board. Finally, report of the trial and results will follow the Consolidated Standards of Reporting Trials (CONSORT) 2010 recommendations.^39^

#### Feasibility of recruitment

In previous studies, we were able to successfully recruit patients within our network of cooperating general practitioners, e.g. in the three-arm GET.FEEDBACK.GP-RCT (depression screening using patient-targeted feedback in general practices).^40^ In addition, resident pulmonologists, our pulmonary out-patient clinic and our psychosomatic out-patient clinic, three self-help groups and patient associations, and social media will support recruitment. The format as an online video consultation and the brevity of the intervention will also facilitate patient enrolment. We expect a high willingness to participate in a psychosocial intervention study for Long COVID symptoms, as patients express high needs in this direction and this area is considered to be massively undersupplied so far.

### Ethics and dissemination

#### Ethical approval

The authors assert that all procedures contributing to this work comply with the ethical standards of the relevant national and institutional committees on human experimentation and with the Helsinki Declaration of 1975, as revised in 2008. All procedures involving human patients were approved by the Local Psychological Ethics Committee at the Center for Psychosocial Medicine of the University Medical Center Hamburg-Eppendorf on 14 February 2022 (approval number LPEK-0446). The trial will further be conducted in accordance with guidelines for Good Clinical Practice, and national and local laws. Before inclusion, eligible participants will provide written informed consent. Data will be stored in the REDCap database in pseudonymised form. A corresponding data management manual and a data review plan will be finalised before recruitment of the first patient. Any changes to the study protocol will be listed in the study registry and publications. The study is registered with the ISRCTN Registry (identifier ISRCTN15068418).

#### Data sharing

In accordance with the ethics committee approval and the DFG guidelines for the handling of research data adopted in 2015, de-identified individual patient data will be made publicly available. Data sharing will be in accordance with the FAIR data principles (Findable, Accessible, Interoperable, and Reusable) and international naming conventions (e.g. Systematised Nomenclature of Medicine) to maximise transparency and scientific reproducibility. According to the WHO Statement on Public Disclosure of Clinical Trials (https://www.who.int/ictrp/reporting-on-findings), the main findings will be submitted for publication in a peer-reviewed journal within 12 months of study completion.

## Results

The results of this study will be published in peer-reviewed journals, presented at national and international conferences, and communicated in lay language to self-help groups and patient associations.

## Discussion

Long COVID is associated with a high burden for those affected, and poses a growing challenge to healthcare systems worldwide. Given that a causal therapy and concrete treatment recommendations are missing so far,^3^ effective and scientifically sound treatment options need to be developed for patients suffering from Long COVID. Study results suggest that illness-related anxiety^2–4^ and dysfunctional symptom expectations^5,6^ play a role in the development of PSS after COVID-19, thus representing a promising therapeutic target for a Long COVID intervention. Since a growing body of research provides evidence that targeted expectation management improves clinical outcomes,^26,27^ we assume that enhancing realistic positive and reducing dysfunctional symptom expectations might also lead to improved clinical outcomes in patients with Long COVID.

To the best of our knowledge, this is the first study to investigate the modifiability of a specific psychological mechanism of symptom persistence in Long COVID. The results of our analyses for hypothesis 1 will allow us to draw conclusions regarding the applicability, potential efficacy and mechanisms of a targeted expectation management intervention for patients with Long COVID. If the effectiveness of the intervention via the proposed mode of action can be proven, it can be used stand-alone or in the context of a broader therapeutic approach, and might thus have an important clinical and potentially socioeconomic impact. The results regarding objective 2 will significantly contribute to a better understanding of symptom persistence in Long COVID, and will shed light on disease-specific risk factors and mechanisms in addition to illness-related anxiety and dysfunctional symptom expectations. Overall, this study will contribute to a better understanding of the contribution of psychological factors to Long COVID symptoms, and thereby advance the development of comprehensive care for patients. Data regarding mechanisms of symptom persistence from the control group will be pooled and compared with data from the SOMACROSS Research Unit.^9^ The results of whether the mechanisms of symptom persistence are disease-specific or also effective across diseases will contribute to the further development of our aetiological model for PSS across diseases.

Although early data on the frequency of Long COVID came from hospitalised cohorts, Long COVID has been shown to occur at very similar rates in patients with mild acute symptoms treated in ambulatory care.^41^ Because of different study designs and assessment times, use of unvalidated measures, lack of inclusion of control groups and focus on individual symptoms instead of Long COVID as a diagnostic entity, the exact prevalence of Long COVID and its natural course are currently difficult to estimate. Nevertheless, the high rate of infected people worldwide and significant number of PSS after a SARS-CoV-2 infection suggest that research into the mechanisms of symptom persistence, as well as treatment options, is of the highest clinical relevance.

One limitation of this study might be its particular focus on illness-related anxiety and dysfunctional symptom expectations as potential risk factors for Long COVID. However, it is inevitable to focus on selected promising constructs, as it is not feasible to investigate all potential risk factors simultaneously. The mere use of self-report instruments is another limitation. Medical aspects, including a history of COVID-19, will be collected from patient information only and will not additionally be verified by patient records or SARS-CoV-2 antibody tests. The fact that fewer people are getting tested for SARS-CoV-2 since the COVID-19 pandemic has progressed and containment measures have been relaxed could lead to a potential sampling bias, with predominantly more patients with longer symptom duration participating in the study.

A challenge in the conduction of the study could be that some patients might be reluctant to consider psychological factors in the pathophysiology of Long COVID, which could potentially result in a lower participation rate or a higher drop-out rate than expected. To counteract this issue, we will collaborate with patient associations and self-help organisations to jointly establish communication strategies. We will also involve affected patients in the development of our expectation management intervention, an aspect that we will include in our study materials (e.g. leaflets). During the intervention, multiple mechanisms in the emergence of Long COVID will be addressed in a sensitive manner, outlining psychological variables as being one of several relevant factors for the development of Long COVID. To enhance comprehensibility, illustrative examples of everyday life will be used carefully in the conveyance of a biopsychosocial model. Finally, as part of screening interviews, potential participants will receive conclusive information about the study before inclusion to prevent drop-outs.

Considering the scientific evidence, the clinical relevance of Long COVID and the high level of suffering of affected patients, the attempt to investigate whether a targeted modification of illness-related anxiety and dysfunctional symptom expectations has a beneficial effect on Long COVID symptoms seems to be a valuable endeavour. Evidence of an effect of modifying illness-related anxiety and dysfunctional symptom expectations on Long COVID symptoms would identify an important mechanism of action, as well as be a promising starting point for future treatments for Long COVID.

## Data Availability

The data that support the findings of this study will be available from the corresponding author, P.E., on reasonable request.
